# Well-mixed plasma and tissue viral populations in RT-SHIV-infected macaques implies a lack of viral replication in the tissues during antiretroviral therapy

**DOI:** 10.1186/s12977-015-0212-2

**Published:** 2015-11-11

**Authors:** Mary F. Kearney, Elizabeth M. Anderson, Charles Coomer, Luke Smith, Wei Shao, Nicholas Johnson, Christopher Kline, Jonathan Spindler, John W. Mellors, John M. Coffin, Zandrea Ambrose

**Affiliations:** HIV Dynamics and Replicaton Program, National Cancer Institute at Frederick, 1050 Boyles Street, Building 535, Room 109, Frederick, MD 21702-1201 USA; Advanced Biomedical Computing Center, SAIC, Frederick, USA; Division of Infectious Diseases, Department of Medicine, University of Pittsburgh School of Medicine, Pittsburgh, PA USA; Department of Molecular Biology and Microbiology, Tufts University, Boston, MA USA

**Keywords:** RT-SHIV, SIV or HIV evolution, HIV diversity, Single genome sequencing (SGS), Nonhuman primate model, Antiretroviral therapy, HIV compartmentalization, HIV persistence, SIV or HIV reservoir

## Abstract

**Background:**

Determining the anatomic compartments that contribute to plasma HIV-1 is critical to understanding the sources of residual viremia during combination antiretroviral therapy (ART). We analyzed viral DNA and RNA populations in the plasma and tissues from macaques infected with SIV containing HIV-1 RT (RT-SHIV) to identify possible sources of persistent viremia and to investigate the effect of ART on viral replication in tissues. Tissues were collected at necropsy from four pigtailed macaques infected for 30 weeks with a diverse population of RT-SHIV. Two animals (6760 and 8232) were untreated and two animals (8030 and 8272) were treated with efavirenz, tenofovir, and emtricitabine for 20 weeks.

**Results:**

A total of 1800 single-genome RT-SHIV *pol* and *env* DNA and RNA sequences were analyzed from the plasma, PBMCs, axillary and mesenteric lymph nodes, spleen, thymus, small intestine, bone marrow, lung, and brain. Analyses of intracellular DNA and RNA populations revealed that the majority of proviruses in tissues from untreated animal 8232 were not expressed, whereas a greater proportion of proviruses in tissues were expressed from 6760. Few intracellular RNA sequences were detected in treated animals and most contained inactivating mutations, such as frame shifts or large deletions. Phylogenetics showed that RT-SHIV DNA populations in tissues were *not* different from virus in contemporary plasma samples in the treated or untreated animals, demonstrating a lack of anatomic compartmentalization and suggesting that plasma viremia is derived from multiple tissue sources. No sequence divergence was detected in the plasma or between tissues in the treated animals after 20 weeks of ART indicating a lack of ongoing replication in tissues during treatment.

**Conclusions:**

Virus populations in plasma and tissues did not differ significantly in either treated or untreated macaques, suggesting frequent exchange of virus or infected cells between tissues and plasma, consistent with non-compartmentalized and widely disseminated infection. There was no genetic evidence of ongoing replication in tissues during suppressive ART.

**Electronic supplementary material:**

The online version of this article (doi:10.1186/s12977-015-0212-2) contains supplementary material, which is available to authorized users.

## Background

Identifying the sources of persistent plasma HIV-1 during treatment with antiretroviral therapy (ART) is critical for designing strategies to target virus producing cells for elimination. Previous studies have attempted to determine whether persistent viremia during successful combination ART results from incomplete suppression of virus replication [1–9] or if it is produced by long-lived and/or proliferating infected cells [2, 9–18]. Our recent study on the genetics of persistent viremia during ART [18] showed that the plasma viral population after long-term ART consists of the same variants that were present prior to initiating ART, indicated that it originated from cells, or the descendants of cells, that were infected prior to therapy. Furthermore, investigations by Bailey et al. and Kearney et al. on the genetics of plasma viremia determined by single-genome sequencing revealed populations of identical viral sequences present during ART [11, 18]. These findings together imply that persistent viremia during ART may be the result of virus release from long-lived, proliferating cells and not from on-going, virus replication. Direct evidence now exists that supports the idea that persistent viremia is maintained by the expression of virus from proliferating blood cells [19]. This study showed clear evidence of proliferation of infected CD4+ T-cells collected from patients at various times before and during therapy. Furthermore, Maldarelli et al. identified a population of proliferating CD4+ cells that carried a viral variant that was also predominant in the plasma of a patient on ART [19]. These findings provide the strongest evidence to date that persistent viremia during ART can arise from expression of HIV-1 from expanded CD4+ cell populations that were infected prior to initiating therapy. A subset of such proliferating cells is likely the reservoir for viral rebound when ART is interrupted and, therefore, must be eliminated to control HIV-1 infection off ART.

The magnitude and the distribution of the HIV reservoir that contributes to viral rebound off ART are incompletely defined. One study (Simonetti et al. CROI 2015) showed that identical HIV-1 sequences found in the plasma of an individual being treated with ART originated from cells in the blood, gut, and lymph nodes that were clonally expanded from a single infected progenitor. These findings suggest that infected, proliferating cells are widely distributed without evidence of compartmentalization, and that persistent viremia during ART may be derived from multiple tissue sources. By contrast, other studies aimed at profiling HIV-1 genetics have concluded that the virus is sometimes compartmentalized across tissues [20–39]. These conflicting results may be explained in part by a study by Bull et al. who showed that the appearance of compartmentalization, in at least some cases, is due to sampling error and/or over-representation of identical viral variants resulting from cell proliferation and not from unique HIV-1 populations existing in different tissues [40]. These findings suggest that infected cells are well distributed across tissues (the brain and genital secretions may be exceptions [41]) and that local expansion of infected cells may influence the frequency of viral variants detected within tissues, giving the false appearance of compartmentalization. A widely distributed and well mixed model of HIV-1 populations would predict that as new variants emerge in tissues, they would be rapidly disseminated through blood to other compartments, as opposed to local compartmentalization of new variants through ongoing replication. If the well mixed model is confirmed, it would have important implications including: (1) plasma virus population would mirror populations in tissues and, as such, could be used to monitor diversity and evolution in tissues, (2) variants arising from ongoing replication in sanctuary tissues sites would be rapidly disseminated to other tissues and readily detectable in the blood, and (3) expanded clones that may be an important reservoir for HIV would be spread throughout tissues.

Additional studies are needed to determine if HIV-1 is well distributed or is compartmentalized across tissues. These studies are challenging to perform in humans because of the difficulty in obtaining relevant human samples for testing. Animal models are an appropriate substitute for such investigations as they enable extensive sampling, including collection at scheduled necropsy, allowing for expansive and detailed analyses of the viral populations in blood and tissues. Animal models also eliminate the complications of inter-host viral diversity and incomplete treatment adherence, which are common issues in human studies. Previous studies of infected macaques have shown that intracellular viral RNA and viral DNA are present in many tissues [42, 43] and that plasma virus is derived from multiple tissue sources such as the gut [44, 45] or from the lymph nodes [46]. To investigate both the anatomic sources of persistent plasma virus during ART, the nature of proliferating cells (marked by expanding viral variants), and the possibility of on-going viral replication in the tissues during ART, we characterized the genetics of viral variants across anatomic sites in untreated and ART-treated pigtailed macaques infected with a strain of SIV_mne_ containing HIV-1 RT (RT-SHIV_mne_) [43, 47, 48]. Infection with RT-SHIV_mne_ has previously been shown to result in pre-therapy RNA levels that are similar to HIV-1 infection in humans, to display dynamics of viral decay on ART similar to that in humans, and to achieve sustained levels of suppression on ART to <30 copies/ml [47, 48]. Additionally, RT-SHIV_mne_ animal models have been shown to be appropriate systems to investigate the viral genetics of persistent viremia [49]. In the current study, we used the RT-SHIV_mne_ macaque model to investigate the sources of plasma viremia, the distribution of identical sequences in tissues, and the possibility of on-going replication in tissues during ART by analyzing single-genome sequences obtained from 11 different anatomic sites in four pigtailed macaques infected with a diverse population of RT-SHIV_mne_. We hypothesized that infected cells would be well dispersed across tissues, making the viral populations indistinguishable from plasma virus and implying multiple anatomic origins of persistent viremia and the lack of on-going replication in tissues [18].

## Results and discussion

### Viral DNA and RNA levels in plasma and tissues from RT-SHIV-infected macaques

We used a subset of samples from four macaques reported in a previous study to have persistent levels of infected cells and RT-SHIV_mne_ expression (hereafter denoted RT-SHIV). As described previously, plasma samples were collected weekly from the 4 pigtailed macaques after inoculation with RT-SHIV and tissue samples were obtained at necropsy 30 weeks post-infection [43]. Two of the animals (6760 and 8232) were left untreated, and two (8272 and 8030) were treated daily with efavirenz, tenofovir, and emtricitabine, beginning 10 weeks post-infection, and continued until sacrifice [43]. As published by Kline et al. plasma viremia was measured weekly or biweekly throughout the original study and showed a thousand-fold difference in set point between the two untreated animals (6760–10^5^ and 8232–10^2^) as well as sustained suppression of viremia <30 copies/mL after 5–10 weeks of ART in both treated animals. DNA and RNA were isolated from necropsy tissues, quantified as previously reported [43] and as described briefly in the methods, and were used for single-genome sequencing in this study (Table 1, Additional file 1: Figure S1). By analyzing data from Kline et al. we found that, despite the thousand-fold difference in plasma RNA levels at the set point in the two untreated animals, the mean difference between levels of RT-SHIV DNA in their tissues was less than 2-fold. This finding suggests that the higher levels of viremia in untreated animal 6760 compared to untreated animal 8232 is more likely due to higher levels of expression of virus from infected cells rather than to a higher frequency of infected cells. This conclusion was confirmed by measuring levels of RT-SHIV RNA in the tissues in these animals, which showed a ratio of 108:1 (RNA:DNA) in animal 6760 and 8:1 in animal 8232. In the two ART-treated animals, RT-SHIV-infected cells were readily detectable but, the levels of viral RNA expression in tissues collected after 30 weeks of treatment were rarely above the limit of the assay detection, indicating that the infected cells that persisted during ART expressed few viral transcripts (Table 1).

**Table 1:** *gag* RNA and DNA copies in tissues at necropsy (week 30/31) and number of single-genome sequences obtained

Tissue	DNA								RNA							
	Untreated				Treated				Untreated				Treated			
					EFV + FTC + TNV								EFV + FTC + TNV			
	6760		8232		8272		8030		6760		8232		8272		8030	
	Copies^a^	# SGS	Copies^a^	# SGS	Copies^a^	# SGS	Copies^a^	# SGS	Copies^b^	# SGS	Copies^b^	# SGS	Copies^b^	# SGS	Copies^b^	# SGS
Plasma	–	–	–	–	–	–	–	**–**	180,000^c^	26	8200^c^	44	<30^c^	7	<30^c^	16
PBMC	271	19	531	27	288	39	52	19	190	27	10	5	<1	11	30	0
Duodenum	64	24	72	33	31	42	<1	3	2040	0	<1	–	<1	6	–	–
Jejunum	36	24	9	29	14	24	19	25	–	–	10	4	<1	0	<1	0
Ileum	330	28	205	23	32	3	<1	0	–	–	270	34	–	–	–	–
Lung	9	23	38	18	9	28	25	0	2990	4	1420	1	<1	0	<1	–
Thymus	7	28	–	–	–	–	200	0	1370	28	–	–	–	–	10	0
Bone marrow	25	21	2	42	4	21	2	12	180	0	10	1	<1	0	<1	0
Spleen	275	25	64	33	52	24	48	16	5670	6	460	0	<1	0	10	0
Axillary LN	833	33	86	24	171	27	32	0	15,680	22	1200	20	<1	1	<1	2
Mesenteric LN	538	27	251	22	629	23	29	23	23,490	39	130	5	<1	0	<1	0
Mean copy number	238		140		137		46		25,734		1171		<1		<9	

– Sample not available

^a^RT-SHIV DNA copies per 10^6^ cells (measured by CCR5 copy number)

^b^RT-SHIV RNA copies per 10^6^ CD4 RNA copies

^c^RT-SHIV RNA copies per ml plasma

### RT-SHIV diversity of the challenge virus and diversity and divergence in animals 1 week post-infection vs. 30 weeks post-infection

**Fig. 1:**
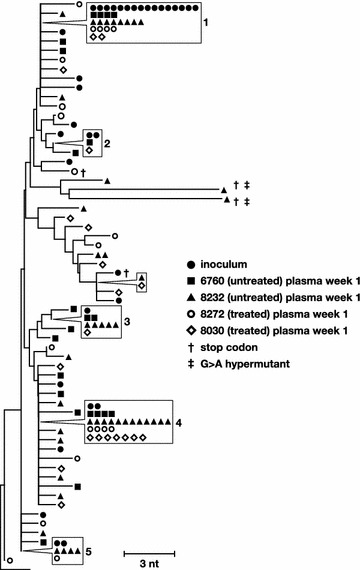
Phylogenetic relationships between single-genome sequences obtained from the challenge virus stock and from the plasma collected at one week post-infection from each animal. G to A hypermutants are marked by *crosses*. *Numbers* refer to clusters of sequences observed more than once here and in subsequent figures. *Symbols* shown in *boxes* indicate identical sequences that are present on the tree at the location indicated by the *pointer*

Single-genome sequencing (SGS) of the RT-SHIV RT coding region was performed on the virus challenge stock and on plasma collected from each animal 1 week post-infection. The viral populations in each sample were analyzed using neighbor-joining trees which are capable of reliably identifying identical variants, a key part our analyses throughout the study (Fig. 1). The RT-SHIV population in the challenge stock was heterogeneous, likely due to its history of serial passage in cell culture [43], and similar sets of variants successfully established infection in all four macaques (Fig. 1). Although the populations in the plasma and the challenge stock were diverse, they did include several dominant variants that could be used to track the seeding and spread of specific variants across the tissues (Fig. 1, numbered 1–5). The similarities of the viral population after inoculation in all four animals imply that differences in viral set point were not related to the diversity of the variants that established infection. The heterogeneity early in infection in these animals resulted in populations that more closely resembled chronic HIV-1 infection in humans than homogeneous acute infections.

**Fig. 2:**
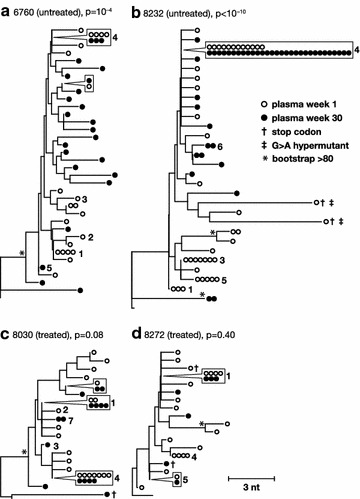
Phylogenetic and panmixia relationships between single-genome plasma sequences obtained one week and 30 weeks post-infection from each animal. Neighbor-joining trees were prepared using consensus B as an out group (*bottom*, unlabeled branch) for the untreated (**a**, **b**) and treated (**c**, **d**) animals. The p values shown next to each animal identifier denote the probability that the virus populations sampled at one week and 30 weeks after infection are not different (panmixia). *Numbers* refer to clusters of sequences observed more than once here and in previous and subsequent figures. *Symbols* shown in *boxes* indicate identical sequences that are present on the tree at the location indicated by the *pointer*. G to A hypermutants are marked by crosses. *Asterisks* indicate bootstrap values >80

In addition to comparing the early plasma diversity to the RT-SHIV challenge virus, we also compared the viral genetic diversity in the plasma at week 1 to the plasma diversity at week 30 in each animal to look for evidence of viral evolution over time by using phylogenetics and a test for panmixia (Fig. 2). The panmixia test is used to determine the probability that two subpopulations are derived from the same, well-mixed population. Due to the large numbers of comparisons between sequences and the possibility of sampling error, pairs of populations with panmixia probabilities of <10^−3^ are considered significantly different [18, 49, 50]. Plasma populations obtained at week 30 were found to be significantly different from week 1 populations in the untreated animals (p = 10^−4^ in animal 6760 and p < 10^−10^ in 8232), but not in the animals treated with ART (p = 0.08 in 8030 and p = 0.4 in 8272) (Fig. 2). We also normalized the data to account for differences in sampling between the treated and untreated animals to be sure that the panmixia test was not influenced by undersampling in the treated animals and found little difference in the panmixia results (p = 0.0005 in animal 6760 and p = 0.0003 in 8232). We accomplished this by generating a script to randomly select 20 sequences from week 1 in the untreated animals (to match the number of sequences in the treated animals at week 1) and to randomly select 7 sequences from week 30 in the untreated animals (to match the number of sequences in the treated animals at week 30), which were then tested for panmixia. This finding implies that significant levels of virus evolution due to replication occurred in the untreated animals over 29 weeks but that there was little or no RT-SHIV replication in the blood of the animals treated with ART, consistent with previous HIV-1 studies in humans [18]. The analyses showed that the change in panmixia in untreated animal 6760 was associated with increasing viral population diversity (0.3–0.5 %), corresponding to the emergence of new variants with longer branches on the trees than the variants present at week 1 (Fig. 2a). Only clones 4 and 5 were still detectable after 30 weeks of infection in animal 6760. By contrast, analysis of samples from untreated 8232 revealed evolution by evidence of a genetic bottleneck in this animal that occurred between week 1 and week 30, selecting especially for clone 4 and resulting in a decrease in viral diversity (0.4–0.2 %) (Fig. 2b). These data show that viral evolution in this model can result from the emergence of new variants, as in animal 6760, or from the selection of existing variants, as in animal 8232. Fewer sequences were obtained from the animals on suppressive ART but the sequences obtained were not phylogenetically different from the population present prior to ART initiation (Fig. 2c, d) indicating that no viral evolution could be detected in blood samples. Two additional clonal variants detected in the phylogenetic analyses in Fig. 2 that are also present in later analyses were labeled 6 and 7 for reference across figures and text.

### Analyses of RT-SHIV proviral populations in multiple tissues of untreated animals

**Fig. 3:**
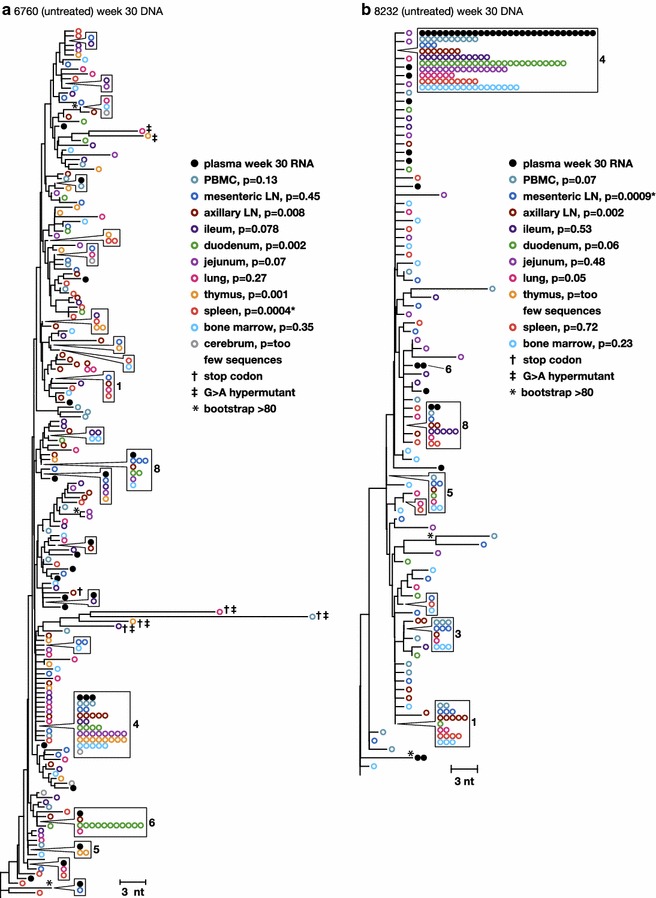
Phylogenetic relationships between single-genome proviral sequences obtained from various anatomical compartments 30 weeks post-infection from the untreated animals **a** 6760 and **b** 8232. The panmixia probabilities comparing virus populations in the tissues (*open colored circles*) with sequences in the plasma (*solid black circles*) are shown next to each tissue in the key. G to A hypermutants are marked by *crosses*. *Numbers* refer to clusters of sequences observed more than once here and in previous and subsequent figures. *Symbols* shown in *boxes* indicate identical sequences that are present on the tree at the location indicated by the *pointer*

The genetics of the RT-SHIV DNA populations were compared across tissues after 30 weeks of infection in the untreated animals and were compared to the plasma virus at week 1 and week 30 to investigate the spread and persistence of viral variants within and between tissues and to investigate the anatomic sources of plasma viremia. The tissues included peripheral blood mononuclear cells (PBMCs), axillary and mesenteric lymph nodes (LN), small intestine (ileum, duodenum, jejunum), lung, thymus, spleen, bone marrow, and brain from one untreated animal (6760). Table 1 shows the number of DNA single-genome sequences obtained from each tissue. DNA populations were compared using (1) average pairwise distance (APD) calculations to measure the genetic diversity within each population (Table 2), (2) the test for panmixia to measure divergence between the tissue populations and the plasma virus at week 1 and week 30 (Table 2; Fig. 3), and (3) neighbor-joining analyses to investigate the clustering of sequences within and between anatomic sites (Fig. 3a, b).

**Table 2:** *pol* population diversity and divergence,between plasma (week 30) and tissues at necropsy (week 30/31)

Tissue	Untreated				Treated			
	6760		8232		8272		8030	
	APD (%)	Panmixia^a^ (19 sequences)	APD (%)	Panmixia^a^ (17 sequences)	APD (%)	Panmixia^a^ (7 sequences)	APD (%)	Panmixia^a^ (12 sequences)
Challenge virus	0.23	**<10** ^−6^*	0.23	**<10** ^−6^*	0.23	0.59	0.23	0.04
Plasma week 1	0.28	**10** ^−4^*	0.36	**<10** ^−6^*	0.3	0.4	0.19	0.08
Plasma week 30	0.5	NA	0.19	NA	0.17	NA	0.35	NA
PBMC	0.85	0.13	0.41	0.07	0.39	0.28	0.26	0.63
Duodenum	0.3	0.002	0.09	0.06	0.32	0.05	0.14	–
Jejunum	0.43	0.07	0.4	0.48	0.41	0.37	0.37	0.3
Ileum	0.59	0.08	0.17	0.53	1.19	–	–	–
Lung	0.82	0.27	0.25	0.06	0.85	0.39	–	–
Thymus	0.46	0.001	–	–	–	–	–	–
Bone marrow	0.47	0.35	0.23	0.24	0.65	0.23	0.33	0.85
Spleen	0.61	0.0004*	0.21	0.72	0.28	0.33	0.19	0.521
Axillary LN	0.5	0.008	0.23	0.002	0.55	0.22	–	–
Mesenteric LN	0.49	0.45	0.39	0.00090*	0.64	0.02	0.24	0.64
Mean APD	0.55		0.26		0.59		0.26	

– Too few sequences to analyze (<7 sequences)

* p value (10^−3^ was used as the criterion for significance due to the numbers of multiple comparisons. Significant differences indicated by bold)

^a^Probability of panmixia of sequences in each tissue compared to an equal number of randomly selected unique sequences in the plasma

A wide range of proviral diversity was found across the tissues (0.09–0.85 %) (Table 2), suggesting that there may be differences among tissue populations. Different tissues or anatomical compartments are likely to exert different selective pressures on viral populations, which could have dramatic effects on viral diversity. Proviral diversities in the tissues of animal 6760 increased relative to the week 1 plasma virus, while tissue diversities in animal 8232 decreased, findings consistent with those in the plasma of the same animals. These changes reflect the emergence of new variants in 6760 and the genetic bottleneck in 8232 and imply that the viral populations in the tissues and plasma virus are well mixed.

Further investigations of sequences from the untreated macaques using the panmixia test showed that although there was a wide range of diversity across the tissues; divergence measured from the plasma virus at the same time point (week 30) was significantly different for only two tissues sites: spleen in 6760 and mesenteric lymph node in 8232 (Table 2; Fig. 3). This finding indicates that the same viral variants are present in plasma and across *most* tissues after 30 weeks of infection and that the wide range in intra-tissue diversity must be due to either sampling error or to different frequencies of the same variants within each tissue compartment.

Neighbor-joining trees confirmed that the vast majority of proviral *pol* sequences obtained from different anatomical compartments were not different from each other or from the plasma virus at the same time point (Fig. 3a, b). Importantly, these data demonstrate that the evolution that occurred between week 1 and week 30 in these animals is reflected across the tissues analyzed, suggesting that these tissue compartments are more likely connected rather than compartmentalized. Single-genome *env* sequences were also obtained from one animal (6760) with the same finding of sequence intermingling across tissues as was found for RT (Additional file 2: Figure S2). Phylogenetic analyses also showed that the variable divergences in two tissues (spleen in 6760 and thymus in 8232) were due to differing frequencies of identical sequences between tissues and plasma; specifically, differences in frequencies of clone 4. Clone 4 was under-represented in the spleen in animal 6760 and in the mesenteric LN in animal 8232 resulting in statistically “divergent” populations. This observation was confirmed by collapsing all identical sequences to single variants, resulting in the loss of significant divergence by panmixia. The different frequencies of these variants across some tissues could be due to sampling error, differential seeding of variants from the challenge virus stock, or from clonal expansion of infected cells. Of note, clones 1, 3, 4, and 5 persisted in the tissues in animal 8232 after 30 weeks of infection whereas these variants (except 4) disappeared from the plasma due to a genetic bottleneck that occurred with the section of clone 4 (only reaching significance in the mesenteric LN). Clones 1, 4, and 5 remained detectable in at least some of the tissues of animal 6760 (Fig. 3a), while only clones 4 and 5 were present in the plasma (reaching significance in the spleen). Only 5 sequences were recovered from the brain tissue samples. All 5 sequences were obtained from the cerebrum of the untreated animal, 6760 with the highest viral set point. Three of the 5 variants matched those found in other tissues while two were unique but closely related (Fig. 3a). These findings show a lack of significant compartmentalization of virus in the brain of this pigtailed macaque, consistent with that observed across other tissues (albeit with limited sampling in this case). This observation is distinct from that in humans where clear evidence for HIV-1 compartmentalization has been shown in cerebral spinal fluid compared to blood in some individuals [29]. In total, the DNA populations in the tissues differed significantly only by the variable frequencies of identical sequences, indicating that viral variants and/or infected cells are well mixed across tissues. Plasma virus contained subpopulations that were not different from the proviral populations in the tissues examined. This finding strongly suggests that viremia is most likely derived from infected cell populations that have spread across most, if not all, tissues in the body.

### Analyses of RT-SHIV intracellular RNA populations in tissues of untreated animals

**Fig. 4:**
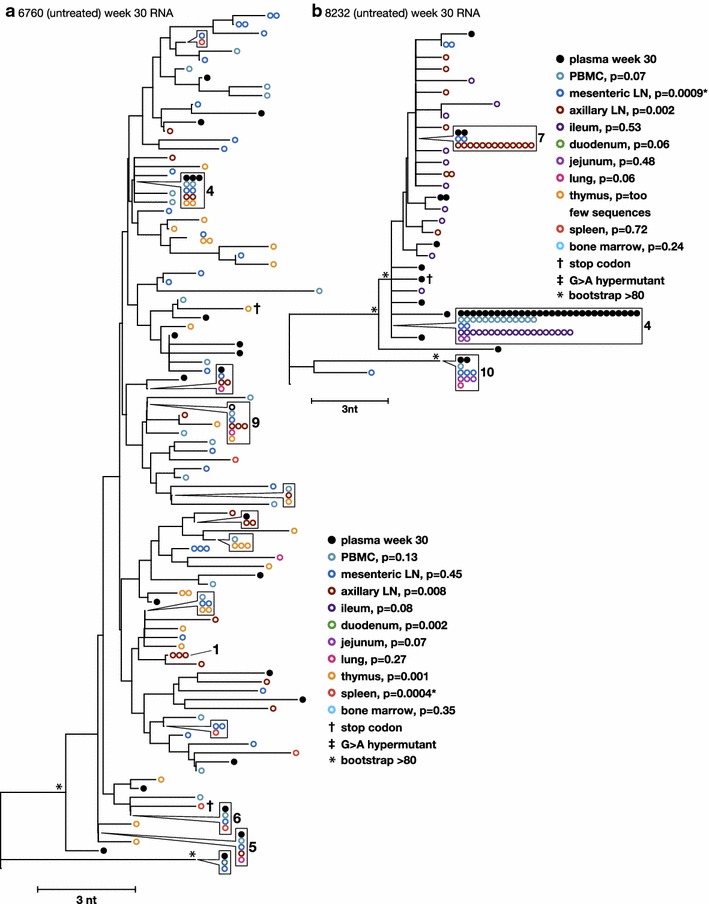
Phylogenetic relationships between intracellular RNA single-genome sequences obtained from different anatomical compartments 30 weeks post-infection from untreated animals 6760 and 8232. *Numbers* refer to clusters of sequences observed more than once here and in previous and subsequent figures. *Symbols* shown in *boxes* indicate identical sequences that are present on the tree at the location indicated by the *pointer*

**Fig. 5:**
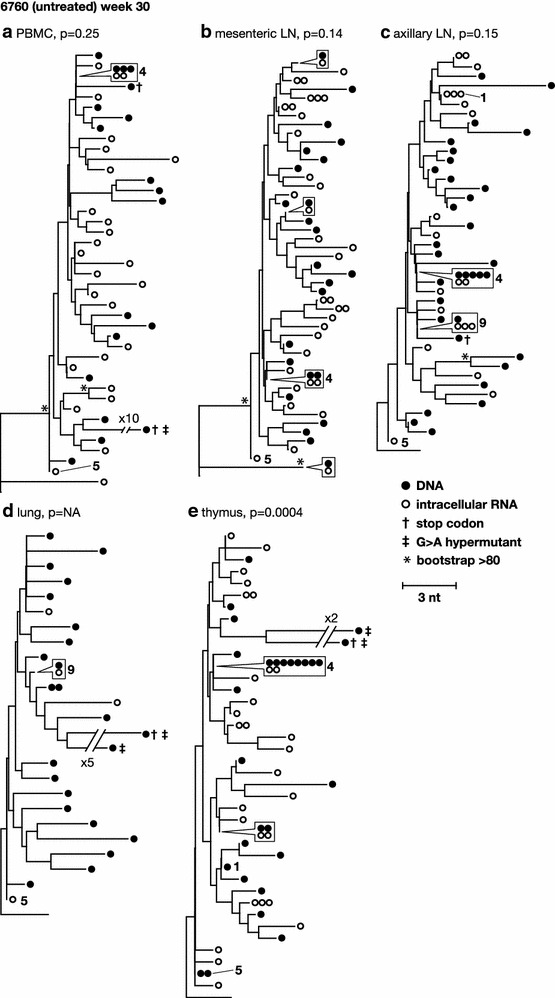
Phylogenetic relationships between intracellular RNA and DNA single-genome sequences obtained from the same anatomical compartments 30 weeks post-infection from untreated animal 6760. p values for panmixia are shown where more than 7 sequences were obtained for both RNA and DNA. *Numbers* refer to clusters of sequences observed more than once here and in previous and subsequent figures. *Symbols* shown in *boxes* indicate identical sequences that are present on the tree at the location indicated by the *pointer*

**Fig. 6:**
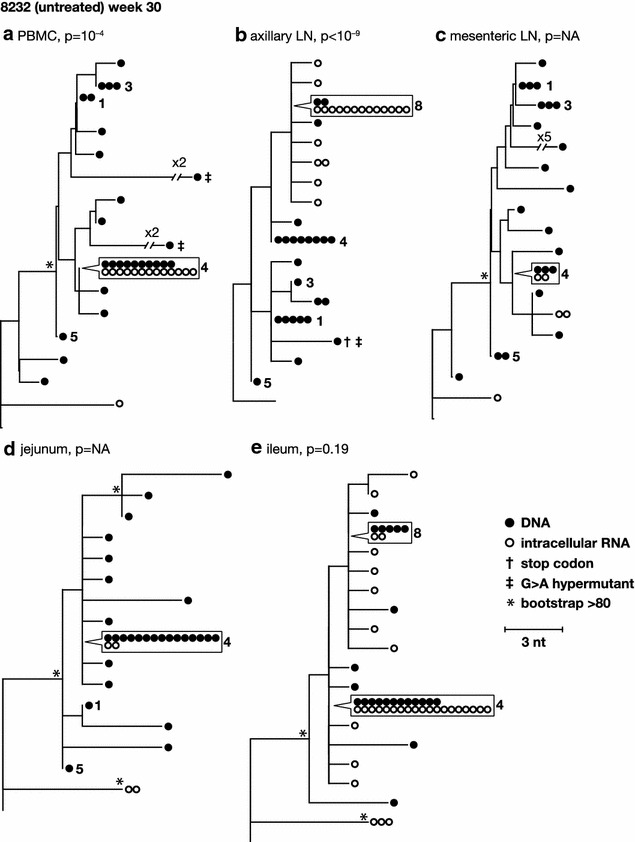
Phylogenetic relationships between intracellular RNA and DNA single-genome sequences obtained from the same anatomical compartments 30 weeks post-infection from untreated animal 8232. p values for panmixia are shown where more than 7 sequences were obtained for both RNA and DNA. *Numbers* refer to clusters of sequences observed more than once here and in previous and subsequent figures. *Symbols* shown in *boxes* indicate identical sequences that are present on the tree at the location indicated by the *pointer*

To characterize the diversity and divergence of RT-SHIV variants expressed in different tissues and to further investigate the sources of plasma viremia, we obtained intracellular viral RNA sequences and compared these populations to those obtained from plasma (Fig. 4) and to the proviral populations from the same tissues (Figs. 5, 6). Table 1 shows the number of RNA sequences obtained from each tissue. Neighbor-joining trees of intracellular RNA sequences obtained across tissues from the untreated animals are shown in Fig. 4. Similar to the viral DNA populations, intracellular RNA variants in the untreated animals intermingled between tissue and plasma sequences and showed no divergence by panmixia (Table 2), revealing a lack of anatomic compartmentalization and a lack of isolated, local evolution or expression in these animals (Fig. 4). These data support our conclusions from the proviral sequence analyses that the infected cells are well mixed across anatomic compartments and that plasma virus could be derived from multiple anatomic sites. We also found that the expressed populations contained clusters of identical sequences (Fig. 4), suggesting either that multiple cells carrying identical variants are expressing viral RNA (likely the case for previously identified clones such as clone 4) or that a single cell per tissue is undergoing high levels of viral expression in comparison to other infected cells (possibly the case for identical sequences that were not found to be clonal in the DNA, such as those labeled 7, 9, and 10 in Fig. 4).

Direct phylogenetic and panmixia comparisons of intracellular RNA and proviral DNA populations from untreated animals 6760 and 8232, including expression from cells carrying identical sequences, are shown in Figs. 5 and 6. Clonal variants were expressed at varying levels in different tissues, ranging from undetectable to high levels of expression. Phylogenetic relationships between intracellular RNA and DNA single-genome sequences obtained from the same anatomical compartments after 30 weeks of infection from untreated animal 6760 showed no structural differences (Fig. 5), indicating that the infected cells expressing viral RNA are diverse, likely contributing to the high level of plasma viremia in this animal. Panmixia p values are shown where more than 7 sequences were obtained for both RNA and DNA and, with one exception, show a lack of significant probability that the RNA population is different from the DNA population (Fig. 5). This finding suggests that, in the absence of ART, a large fraction of the infected cells across all tissues appear to be expressing viral RNAs. By contrast, phylogenetic analyses of intracellular RNA and DNA from the same anatomical compartments after 30 weeks of infection in untreated animal 8232 with a low viral set point (1000-fold lower than in animal 6760) showed a significant difference in panmixia in some of tissues analyzed (Fig. 6). The difference was primarily due to dominance of viral expression from clonal proviruses 4 and 8, shown in the trees as RNA sequences matching the DNA variants and identified with their clonal reference numbers. This finding suggests that a smaller proportion of infected cells were expressing RT-SHIV RNA in this animal compared to animal 6760, consistent with the lower level of plasma viremia in animal 8232. Analyses of intracellular RNA populations revealed that the majority of proviruses in tissues from 8232 were not likely expressed, whereas tissues from 6760 had a greater proportion of proviruses being expressed. These data suggested two important findings: (1) compartmentalization of proviral populations in the tissues compared to viral populations in the blood was only very rarely statistically significant, and (2) when rare compartmentalized proviral populations did occur, the variants that were expressed were not different from the virus population in plasma. These findings are important because they imply that exchange of virus and/or infected cells between the blood and the tissues may be frequent and that viral evolution may not occur in isolation in the tissues in the animal model studied. Although local viral spread may occur, it appears to be in tandem with the mixing of variants between all the tissues and the blood.

### Analyses of RT-SHIV proviral populations in multiple tissues of animals treated with ART

**Fig. 7:**
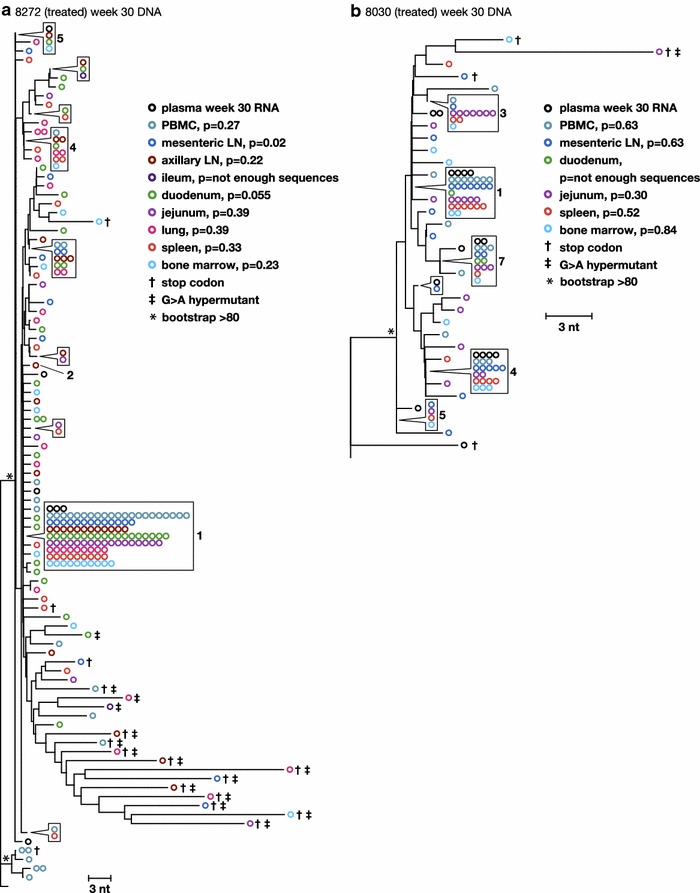
Phylogenetic relationships between single-genome proviral sequences obtained from various anatomical compartments after 20 weeks of combination ART from animals **a** 8272 and **b** 8030. The panmixia probabilities comparing virus populations in the tissues (*open colored circles*) with sequences in the plasma (*open black circles*) are shown next to each tissue in the key. G to A hypermutants are marked by *crosses*. *Numbers* refer to clusters of sequences observed more than once here and in previous and subsequent figures. *Symbols* shown in *boxes* indicate identical sequences that are present on the tree at the location indicated by the *pointer*. *Asterisks* indicate bootstrap values >80

The genetics of the RT-SHIV DNA populations were compared across tissues and to the plasma virus at week 1 and week 30 in the two macaques treated for 20 weeks with combination ART to investigate the spread and persistence of viral variants within and between tissues and to investigate the anatomic sources of persistent viremia during ART. Table 1 shows the number of DNA single-genome sequences obtained from each tissue in the two treated macaques, 8030 and 8272. DNA populations were compared using (1) APD calculations to measure the genetic diversity within each population (Table 2), (2) the test for panmixia to measure divergence between the tissue populations and between tissues and plasma (Table 2), and (3) neighbor-joining analyses to investigate the clustering of sequences within and between anatomic sites and with plasma virus (Fig. 7a, b).

**Fig. 8:**
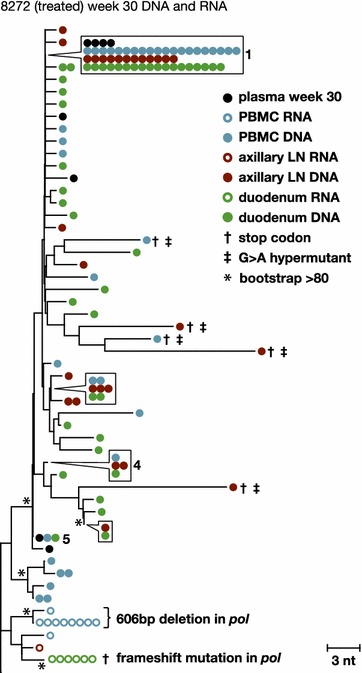
Phylogenetic relationships between intracellular RNA and DNA single-genome sequences obtained from the same anatomical compartments after 20 weeks of combination ART from animal 8272. p values for panmixia are shown where more than 7 sequences were obtained for both RNA and DNA. *Numbers* refer to clusters of sequences observed more than once here and in previous and subsequent figures. *Symbols* shown in *boxes* indicate identical sequences that are present on the tree at the location indicated by the *pointer*

As in the untreated animals, a wide range of proviral diversity was found across the tissues (0.14–1.19 %) with no overall reduction after 20 weeks of therapy (Table 2). This finding suggests that there may be differences among the populations between tissues (maybe due to different frequencies of G to A hypermutants) and that this distribution is not affected by short-term combination ART. A lack of change in viral diversity with short-term therapy is consistent with our previous observation of plasma virus in HIV-infected humans on ART [18] and in RT-SHIV-infected macaques on suppressive ART [18]. Further investigation of sequences from the treated macaques using the panmixia test showed that, although there was a wide range of diversities across the tissues, the populations were not significantly divergent from each other or from the plasma virus at the same time point (Table 2; Fig. 8). The panmixia results suggest a lack of compartmentalization across the tissues.

Neighbor-joining trees revealed that proviral *pol* sequences obtained from different anatomical compartments intermingled among themselves, consistent with the panmixia results of no divergence, and among sequences obtained from plasma at the same time points (Fig. 8). The finding that the DNA populations in the tissues differed only by the variable frequencies of identical sequences, especially for the numbered clones, indicates that viral variants and/or infected cells are well mixed among all tissues analyzed. The differing frequencies of identical sequences are likely due to varying levels of persistence and/or proliferation of infected cells. Clone 1 remained dominant in the plasma from both treated animals, as it was in the virus challenge stock, but it did not persist in the untreated animals. Plasma virus collected during ART contained subpopulations that were not different from the proviral populations in the tissues examined, including the gastrointestinal tract, which has been proposed to be a sanctuary site for virus replication during ART [32, 51]. This finding suggests that persistent viremia during ART is most likely derived from multiple anatomic sites.

### Analyses of RT-SHIV intracellular RNA populations in tissues of animals treated with ART

To characterize the diversity and divergence of RT-SHIV variants expressed in different tissues and to further investigate the sources of persistent plasma viremia during ART, we sequenced viral intracellular RNA variants and compared these populations to those in the plasma and to the proviral populations in the tissues of ART-treated animal 8272 (Fig. 7). The same analysis could not be done for animal 8030 due to the low numbers of intracellular RNA sequences obtained. Table 1 shows the number of RNA sequences obtained from each tissue from both treated macaques. In contrast to the untreated macaques, few intracellular RNA sequences were detected in animals treated with ART and those that were detected contained frame shift mutations or large deletions, indicating that they were not replication competent. Furthermore, these mutant RNAs did not cluster with plasma virus or the proviral DNA detected in the tissues. This finding suggests that a minority of the infected cells that persist during ART express viral RNA and those RNAs that are actively expressed are frequently defective (Fig. 7), explaining why only very low levels of plasma viremia persist during ART. Most likely, the majority of productively expressing RT-SHIV-infected cells die within 20 weeks of treatment with combination ART, leaving primarily latently infected cells or cells that contain defective proviruses. Although all the expressed RNA sequences detected in the treated animals were defective, no defects were found in the genomes of virus that persisted at very low levels in plasma during ART in these animals, revealing that some infected cells expressing variants that may be replication competent also persist during ART but at levels below the detection limit of our sampling. Similarly, replication competent HIV-1 variants have been reported to persist in HIV-infected humans treated with ART [52], and are likely to be present at some level in most if not all treated patients [53]. In addition to the expressed variants being defective, we also found that the expressed populations contained clusters of identical sequences (Fig. 7). Populations of identical intracellular RNA sequences suggest either that multiple cells carrying identical variants were expressing viral RNA or that single cells (or their descendants) were undergoing high levels of viral expression in comparison to other infected cells. The finding that the identical variants expressed during ART were all defective implies that either their expression is from a proliferating cell population or from a single cell, but not from local spread through viral replication.

## Conclusions

The reservoir for HIV-1 during ART must consist of cells containing replication competent proviruses that persist and sometimes proliferate despite therapy and result in rebound viremia when ART is stopped. The number, identity, distribution, and expression levels of these persisting cells are currently unknown. Our findings show that the reservoir for RT-SHIV likely includes infected cells that are well mixed in different anatomic sites and that plasma virus may be derived from multiple tissue compartments. These findings are important because they imply that exchange of virus and/or infected cells between the blood and many tissues is frequent and that viral evolution does not likely occur and persist in isolation in any specific tissues investigated here, including but not limited to PBMC, lymph nodes, gastrointestinal tract, and spleen. Furthermore, the results of this study show that genetic analyses on plasma virus appear to reflect what is taking place in the tissues in both the untreated and treated animals. Consequently, if viral replication during ART is on-going in the gut, as has been proposed, we would expect the newly emergent variants to be detectable in the plasma during highly suppressive ART. Such an occurrence was not found here at the level of detection provided by single-genome sequencing. Lastly, we found RNA expression of defective proviruses in both treated and untreated macaques as well as a possible correlation between the levels proviral expression and plasma virus load in the untreated animals. In conclusion, these studies strongly suggest that the reservoir for RT-SHIV_mne_ during ART is broadly disseminated throughout the tissues, that infected cells persisting and/or proliferating during ART only rarely express viral products and, when they do, the genomes are often defective, and that the genetics of residual viremia during ART reflect the state of expression in the gut and lymphoid tissues as well as the blood.

## Methods

### Virus

RT-SHIV_mne_ is a pathogenic SIV/HIV chimeric virus in which SIV_mne_ RT is replaced by HIV-1_HxB2_ RT [48]. The challenge stock was grown in CEMx174 cells and was titered on TZM-bl cells. The virus was previously shown to be replication competent and pathogenic in pigtailed macaques (*Macaca nemestrina*) as a useful in vivo model to study antiretroviral suppression and emergence of drug resistance [47].

### Animals

Blood and tissue samples were obtained from pigtailed macaques infected for a study previously published [43]. Briefly, male pigtailed macaques were infected intravenously with approximately 1 × 10^5^ infectious units of RT-SHIV_mne_. All animals were housed at the National Institutes of Health (NIH) in accordance with the American Association of Accreditation of Laboratory Animal Care standards and all procedures were performed according to protocols approved by the Institutional Animal Care and Use Committee of the National Cancer Institute. Two animals were left untreated for 30 weeks post-infection and two received daily ART beginning at 10 weeks of infection until necropsy: 20 mg/kg tenofovir and 40 mg/kg emtricitabine were administered subcutaneously and 400 mg EFV was administered orally in treats. Blood was drawn weekly or biweekly, from which plasma and PBMC were separated. At week 30 post-infection, animals were euthanized and necropsies were performed as previously described [43].

### Viral and host cell DNA and RNA isolation and measurement in PBMC and tissues

Virus was pelleted from EDTA-anticoagulated plasma taken at each time point for each animal and quantitative RT-PCR (qRT-PCR) was performed to determine the number SIV RNA (gag) copy equivalents per ml (copy Eq/ml) of plasma essentially as previously described (Cline et al. 2005). The assay limit for quantitation was 30 copy Eq/ml of plasma. Total DNA (viral and genomic) was extracted from tissues with lysis buffer (Nuclei lysis solution, Promega, Madison, WI, USA) after extensive washing using a TissueLyser (Qiagen, Valencia, CA, USA). Tissues were processed and nucleic acids extracted as previously described [43]. Briefly, tissue DNA was extracted using the Wizard genomic purification kit (Promega) and PBMC DNA was extracted using the Blood DNA kit (Qiagen). qPCR *(gag)* assays were performed as described previously with a limit of detection of 1 copy/sample [43].

Viral and host cell RNA were extracted from tissues stored in RNAlater. Tissues were rinsed with nuclease-free water and homogenized using a TissueLyser in the presence of lysis buffer (RTL, Qiagen) and 20 U of RNase inhibitor (Ambion). RNA was extracted from lymphoid tissues and lung using the RNeasy kit (Qiagen). As this method did not yield good RNA recovery from gastrointestinal tract and brain tissues, TRI Reagent (Sigma-Aldrich, St. Louis, MO, USA) was used to extract RNA from brain and gut tissues, as previously described (10) [54]. Purified RNA was treated with 10U RNase-free DNase I (Roche, Indianapolis, IN, USA) and then precipitated and washed again using the RNeasy kit. qRT-PCR assays were performed as previously described [43].

### Single-genome sequencing and genetic analyses

SGS of HIV-1 *pol* was performed as previously described [49]. Sequences were aligned using ClustalW. Population genetic diversity was calculated as average pairwise difference (APD) using MEGA4 (http://www.megasoftware.net) and an in-house program [55]. Shifts in population structure were calculated using a subdivision test for panmixia with a significance cut off level of p < 10^−3^ as described by the original report to account for the high number of comparisons between sequences and nucleotide sites (1, 34, 23). The probability of 10^−3^ for assigning a significant change in viral populations obtained from single-genome sequencing was derived statistically taking into consideration that every nucleotide position is compared in every two possible sets of sequences. This approach results in more than 10^12^ comparisons between populations of only 10 sequences. Neighbor-joining phylogenetic analyses were done using MEGA4. Trees were rooted on the subtype B consensus sequence (http://www.HIV-1.lanl.gov) shown as the lowest (unmarked) branch of each tree.

## Additional files

10.1186/s12977-015-0212-2 RT-SHIV RNA copies detected by qRT-PCR in (A) plasma or (B) tissues of RT-SHIV infected animals at time of necropsy. The limit of detection of plasma viral RNA is 30 copies Eq/ml, indicated by the dashed line. The limit of detection of tissue viral RNA is 1 copy and was normalized per 10^6^ CD4 copies. *indicates below the limit of detection; NA indicates that the sample was not available.
10.1186/s12977-015-0212-2 Phylogenetic relationships between single-genome proviral env sequences obtained from various anatomical compartments 30 weeks post-infection from the untreated animal 6760. The *env* sequence populations in the tissues (open colored circles) were not significantly different from each other indicating that the virus is well circulated across compartments, consistent with the results from the pol analyses. Highly divergent sequences are G to A hypermutants.

## Abbreviations

ART: antiretroviral therapy
DNA: deoxyribonucleic acid
RNA: ribonucleic acid
qPCR: quantitative polymerase chain reaction
RT: reverse transcriptase
RT-SHIV: reverse transcriptase-simian/human immunodeficiency virus
SGS: single-genome sequencing

## References

[1] Shiu C, Cunningham CK, Greenough T, Muresan P, Sanchez-Merino V, Carey V, Jackson JB, Ziemniak C, Belzer M, Ray SC, Luzuriaga K, Persaud D, Pediatric AIDS Clinical Trials Group P1059 Team (2009). Identification of ongoing HIV-1 replication in residual viremia during recombinant HIV-1 poxvirus immunizations in patients with clinically undetectable viral loads on durable suppressive HAART. J Virol.

[2] Chun TW, Nickle DC, Justement JS, Meyers JH, Roby G, Hallahan CW, Kottilil S, Moir S, Mican JM, Mullins JI (2008). Persistence of HIV in gut-associated lymphoid tissue despite long-term antiretroviral therapy. J Infect Dis.

[3] Gunthard HF, Wong JK, Ignacio CC, Guatelli JC, Riggs NL, Havlir DV, Richman DD (1998). Human immunodeficiency virus replication and genotypic resistance in blood and lymph nodes after a year of potent antiretroviral therapy. J Virol.

[4] Benito JM, Lopez M, Lozano S, Martinez P, Gonzalez-Lahoz J, Soriano V (2004). CD38 expression on CD8 T lymphocytes as a marker of residual virus replication in chronically HIV-infected patients receiving antiretroviral therapy. AIDS Res Hum Retroviruses.

[5] Cohen Stuart JW, Hazebergh MD, Hamann D, Otto SA, Borleffs JC, Miedema F, Boucher CA, de Boer RJ (2000). The dominant source of CD4+ and CD8+ T-cell activation in HIV infection is antigenic stimulation. J Acquir Immune Defic Syndr.

[6] Martinez E, Arnedo M, Giner V, Gil C, Caballero M, Alos L, Garcia F, Holtzer C, Mallolas J, Miro JM (2001). Lymphoid tissue viral burden and duration of viral suppression in plasma. Aids.

[7] Ruiz L, van Lunzen J, Arno A, Stellbrink HJ, Schneider C, Rull M, Castella E, Ojanguren I, Richman DD, Clotet B (1999). Protease inhibitor-containing regimens compared with nucleoside analogues alone in the suppression of persistent HIV-1 replication in lymphoid tissue. AIDS.

[8] Martinez MA, Cabana M, Ibanez A, Clotet B, Arno A, Ruiz L (1999). Human immunodeficiency virus type 1 genetic evolution in patients with prolonged suppression of plasma viremia. Virology.

[9] Josefsson L, von Stockenstrom S, Faria NR, Sinclair E, Bacchetti P, Killian M, Epling L, Tan A, Ho T, Lemey P (2013). The HIV-1 reservoir in eight patients on long-term suppressive antiretroviral therapy is stable with few genetic changes over time. Proc Natl Acad Sci USA.

[10] Maldarelli F, Palmer S, King MS, Wiegand A, Polis MA, Mican J, Kovacs JA, Davey RT, Rock-Kress D, Dewar R (2007). ART suppresses plasma HIV-1 RNA to a stable set point predicted by pretherapy viremia. PLoS Pathog.

[11] Bailey JR, Sedaghat AR, Kieffer T, Brennan T, Lee PK, Wind-Rotolo M, Haggerty CM, Kamireddi AR, Liu Y, Lee J (2006). Residual human immunodeficiency virus type 1 viremia in some patients on antiretroviral therapy is dominated by a small number of invariant clones rarely found in circulating CD4+ T cells. J Virol.

[12] Palmer S, Maldarelli F, Wiegand A, Bernstein B, Hanna GJ, Brun SC, Kempf DJ, Mellors JW, Coffin JM, King MS (2008). Low-level viremia persists for at least 7 years in patients on suppressive antiretroviral therapy. Proc Natl Acad Sci USA.

[13] Dinoso JB, Kim SY, Wiegand AM, Palmer SE, Gange SJ, Cranmer L, O’Shea A, Callender M, Spivak A, Brennan T (2009). Treatment intensification does not reduce residual HIV-1 viremia in patients on highly active antiretroviral therapy. Proc Natl Acad Sci USA.

[14] Shen L, Siliciano RF (2008). Viral reservoirs, residual viremia, and the potential of highly active antiretroviral therapy to eradicate HIV infection. J Allergy Clin Immunol.

[15] Siliciano RF (2005). Scientific rationale for antiretroviral therapy in 2005: viral reservoirs and resistance evolution. Top HIV Med.

[16] Kieffer TL, Finucane MM, Nettles RE, Quinn TC, Broman KW, Ray SC, Persaud D, Siliciano RF (2004). Genotypic analysis of HIV-1 drug resistance at the limit of detection: virus production without evolution in treated adults with undetectable HIV loads. J Infect Dis.

[17] Fourati S, Lambert-Niclot S, Soulie C, Malet I, Valantin MA, Descours B, Ait-Arkoub Z, Mory B, Carcelain G, Katlama C (2012). HIV-1 genome is often defective in PBMCs and rectal tissues after long-term HAART as a result of APOBEC3 editing and correlates with the size of reservoirs. J Antimicrob Chemother.

[18] Kearney MF, Spindler J, Shao W, Yu S, Anderson EM, O’Shea A, Rehm C, Poethke C, Kovacs N, Mellors JW (2014). Lack of detectable HIV-1 molecular evolution during suppressive antiretroviral therapy. PLoS Pathog.

[19] Maldarelli F, Wu X, Su L, Simonetti FR, Shao W, Hill S, Spindler J, Ferris AL, Kearney JW, Coffin MF, Carcelain JM, Hughes SH (2014). Specific HIV integration sites are linked to clonal expansion and persistence of infected cells. Science.

[20] Fourati S, Lambert-Niclot S, Soulie C, Wirden M, Malet I, Valantin MA, Tubiana R, Simon A, Katlama C, Carcelain G (2014). Differential impact of APOBEC3-driven mutagenesis on HIV evolution in diverse anatomical compartments. Aids.

[21] Rozera G, Abbate I, Vlassi C, Giombini E, Lionetti R, Selleri M, Zaccaro P, Bartolini B, Corpolongo A, D’Offizi G (2014). Quasispecies tropism and compartmentalization in gut and peripheral blood during early and chronic phases of HIV-1 infection: possible correlation with immune activation markers. Clin Microbiol Infect Off Publ Eur Soc Clin Microbiol Infect Dis.

[22] Penton PK, Blackard JT (2014). Analysis of HIV quasispecies suggests compartmentalization in the liver. AIDS Res Hum Retroviruses.

[23] Stam AJ, Nijhuis M, van den Bergh WM, Wensing AM (2013). Differential genotypic evolution of HIV-1 quasispecies in cerebrospinal fluid and plasma: a systematic review. AIDS Rev.

[24] Pou C, Codoner FM, Thielen A, Bellido R, Perez-Alvarez S, Cabrera C, Dalmau J, Curriu M, Lie Y, Noguera-Julian M (2013). HIV-1 tropism testing in subjects achieving undetectable HIV-1 RNA: diagnostic accuracy, viral evolution and compartmentalization. PLoS One.

[25] Falcone EL, Adegbulugbe AA, Sheikh V, Imamichi H, Dewar RL, Hammoud DA, Sereti I, Lane HC (2013). Cerebrospinal fluid HIV-1 compartmentalization in a patient with AIDS and acute varicella-zoster virus meningomyeloradiculitis. Clin Infect Dis Off Publ Infect Dis Soc Am.

[26] Blackard JT (2012). HIV compartmentalization: a review on a clinically important phenomenon. Curr HIV Res.

[27] Heath L, Fox A, McClure J, Diem K, van’t Wout AB, Zhao H, Park DR, Schouten JT, Twigg HL, Corey L, et al. Evidence for limited genetic compartmentalization of HIV-1 between lung and blood. PLoS One. 2009;4:e6949. 10.1371/journal.pone.0006949 PMC2736399 19759830

[28] Reeve AB, Patel K, Pearce NC, Augustus KV, Domingues HG, O’Neil SP, Novembre FJ (2009). Reduced genetic diversity in lymphoid and central nervous system tissues and selection-induced tissue-specific compartmentalization of neuropathogenic SIVsmmFGb during acute infection. AIDS Res Hum Retroviruses.

[29] Harrington PR, Schnell G, Letendre SL, Ritola K, Robertson K, Hall C, Burch CL, Jabara CB, Moore DT, Ellis RJ (2009). Cross-sectional characterization of HIV-1 env compartmentalization in cerebrospinal fluid over the full disease course. AIDS.

[30] Diem K, Nickle DC, Motoshige A, Fox A, Ross S, Mullins JI, Corey L, Coombs RW, Krieger JN (2008). Male genital tract compartmentalization of human immunodeficiency virus type 1 (HIV). AIDS Res Hum Retroviruses.

[31] Caragounis EC, Gisslen M, Lindh M, Nordborg C, Westergren S, Hagberg L, Svennerholm B (2008). Comparison of HIV-1 pol and env sequences of blood, CSF, brain and spleen isolates collected ante-mortem and post-mortem. Acta Neurol Scand.

[32] van Marle G, Gill MJ, Kolodka D, McManus L, Grant T, Church DL (2007). Compartmentalization of the gut viral reservoir in HIV-1 infected patients. Retrovirology.

[33] Harrington PR, Connell MJ, Meeker RB, Johnson PR, Swanstrom R (2007). Dynamics of simian immunodeficiency virus populations in blood and cerebrospinal fluid over the full course of infection. J Infect Dis.

[34] Philpott S, Burger H, Tsoukas C, Foley B, Anastos K, Kitchen C, Weiser B (2005). Human immunodeficiency virus type 1 genomic RNA sequences in the female genital tract and blood: compartmentalization and intrapatient recombination. J Virol.

[35] Smit TK, Brew BJ, Tourtellotte W, Morgello S, Gelman BB, Saksena NK (2004). Independent evolution of human immunodeficiency virus (HIV) drug resistance mutations in diverse areas of the brain in HIV-infected patients, with and without dementia, on antiretroviral treatment. J Virol.

[36] Petito CK (2004). Human immunodeficiency virus type 1 compartmentalization in the central nervous system. J Neurovirol.

[37] Ohagen A, Devitt A, Kunstman KJ, Gorry PR, Rose PP, Korber B, Taylor J, Levy R, Murphy RL, Wolinsky SM, Gabuzda D (2003). Genetic and functional analysis of full-length human immunodeficiency virus type 1 env genes derived from brain and blood of patients with AIDS. J Virol.

[38] Kemal KS, Foley B, Burger H, Anastos K, Minkoff H, Kitchen C, Philpott SM, Gao W, Robison E, Holman S (2003). HIV-1 in genital tract and plasma of women: compartmentalization of viral sequences, coreceptor usage, and glycosylation. Proc Natl Acad Sci USA.

[39] Korber BT, Kunstman KJ, Patterson BK, Furtado M, McEvilly MM, Levy R, Wolinsky SM (1994). Genetic differences between blood- and brain-derived viral sequences from human immunodeficiency virus type 1-infected patients: evidence of conserved elements in the V3 region of the envelope protein of brain-derived sequences. J Virol.

[40] Bull ME, Heath LM, McKernan-Mullin JL, Kraft KM, Acevedo L, Hitti JE, Cohn SE, Tapia KA, Holte SE, Dragavon JA (2013). Human immunodeficiency viruses appear compartmentalized to the female genital tract in cross-sectional analyses but genital lineages do not persist over time. J Infect Dis.

[41] Swanstrom R, Coffin J (2012). HIV-1 pathogenesis: the virus. Cold Spring Harb Perspect Med.

[42] North TW, Higgins J, Deere JD, Hayes TL, Villalobos A, Adamson L, Shacklett BL, Schinazi RF, Luciw PA (2010). Viral sanctuaries during highly active antiretroviral therapy in a nonhuman primate model for AIDS. J Virol.

[43] Kline C, Ndjomou J, Franks T, Kiser R, Coalter V, Smedley J, Piatak M, Mellors JW, Lifson JD, Ambrose Z (2013). Persistence of viral reservoirs in multiple tissues after antiretroviral therapy suppression in a macaque RT-SHIV model. PLoS One.

[44] Petravic J, Vanderford TH, Silvestri G, Davenport M (2013). Estimating the contribution of the gut to plasma viral load in early SIV infection. Retrovirology.

[45] Lay MD, Petravic J, Gordon SN, Engram J, Silvestri G, Davenport MP (2009). Is the gut the major source of virus in early simian immunodeficiency virus infection?. J Virol.

[46] Rothenberger MK, Keele BF, Wietgrefe SW, Fletcher CV, Beilman GJ, Chipman JG, Khoruts A, Estes JD, Anderson J, Callisto SP (2015). Large number of rebounding/founder HIV variants emerge from multifocal infection in lymphatic tissues after treatment interruption. Proc Natl Acad Sci USA.

[47] Ambrose Z, Palmer S, Boltz VF, Kearney M, Larsen K, Polacino P, Flanary L, Oswald K, Piatak M, Smedley J (2007). Suppression of viremia and evolution of human immunodeficiency virus type 1 drug resistance in a macaque model for antiretroviral therapy. J Virol.

[48] Ambrose Z, Boltz V, Palmer S, Coffin JM, Hughes SH, Kewalramani VN (2004). In vitro characterization of a simian immunodeficiency virus-human immunodeficiency virus (HIV) chimera expressing HIV type 1 reverse transcriptase to study antiviral resistance in pigtail macaques. J Virol.

[49] Kearney M, Spindler J, Shao W, Maldarelli F, Palmer S, Hu SL, Lifson JD, KewalRamani VN, Mellors JW, Coffin JM, Ambrose Z (2011). Genetic diversity of simian immunodeficiency virus encoding HIV-1 reverse transcriptase persists in macaques despite antiretroviral therapy. J Virol.

[50] Achaz G, Palmer S, Kearney M, Maldarelli F, Mellors JW, Coffin JM, Wakeley J (2004). A robust measure of HIV-1 population turnover within chronically infected individuals. Mol Biol Evol.

[51] Patterson KB, Prince HA, Stevens T, Shaheen NJ, Dellon ES, Madanick RD, Jennings S, Cohen MS, Kashuba AD (2013). Differential penetration of raltegravir throughout gastrointestinal tissue: implications for eradication and cure. AIDS.

[52] Ho YC, Shan L, Hosmane NN, Wang J, Laskey SB, Rosenbloom DI, Lai J, Blankson JN, Siliciano JD, Siliciano RF (2013). Replication-competent noninduced proviruses in the latent reservoir increase barrier to HIV-1 cure. Cell.

[53] Kim M, Hosmane NN, Bullen CK, Capoferri A, Yang HC, Siliciano JD, Siliciano RF (2014). A primary CD4(+) T cell model of HIV-1 latency established after activation through the T cell receptor and subsequent return to quiescence. Nat Protoc.

[54] Chomczynski P, Mackey K (1995). Short technical reports. Modification of the TRI reagent procedure for isolation of RNA from polysaccharide- and proteoglycan-rich sources. Biotechniques.

[55] Kearney M, Maldarelli F, Shao W, Margolick JB, Daar ES, Mellors JW, Rao V, Coffin JM, Palmer S (2009). Human immunodeficiency virus type 1 population genetics and adaptation in newly infected individuals. J Virol.

