# Supplementary figures and images for: Well-mixed plasma and tissue viral populations in RT-SHIV-infected macaques implies a lack of viral replication in the tissues during antiretroviral therapy
Source: Retrovirology. 2015 Nov 11;12:93. doi: 10.1186/s12977-015-0212-2 (PMC4642622; doi:10.1186/s12977-015-0212-2)

KEARNEY - Supplemental Figure 01

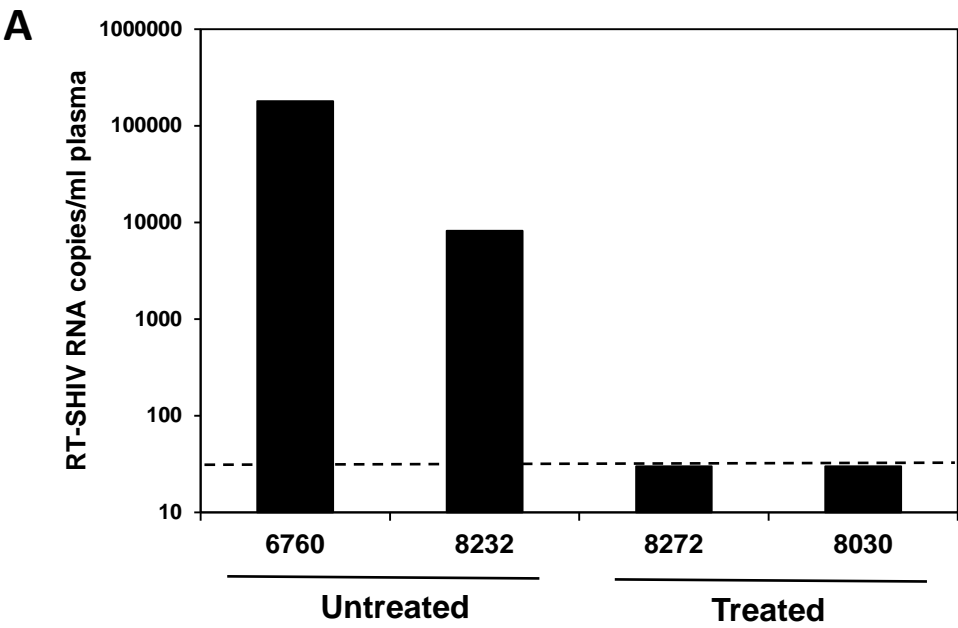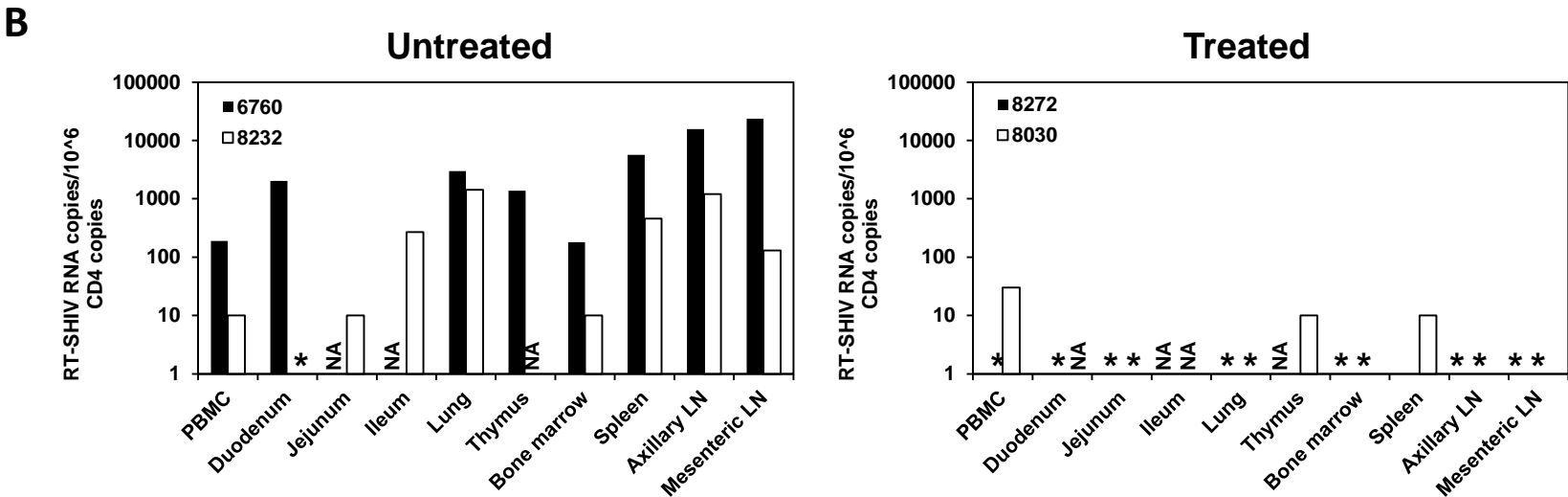

Supplement: Supplementary file 1 — 10.1186/s12977-015-0212-2 RT-SHIV RNA copies detected by qRT-PCR in (A) plasma or (B) tissues of RT-SHIV infected animals at time of necropsy. The limit of detection of plasma viral RNA is 30 copies Eq/ml, indicated by the dashed line. The limit of detection of tissue viral RNA is 1 copy and was normalized per 106 CD4 copies. *indicates below the limit of detection; NA indicates that the sample was not available. [file 12977_2015_212_MOESM1_ESM.pdf]

KEARNEY—Supplemental Figure 02

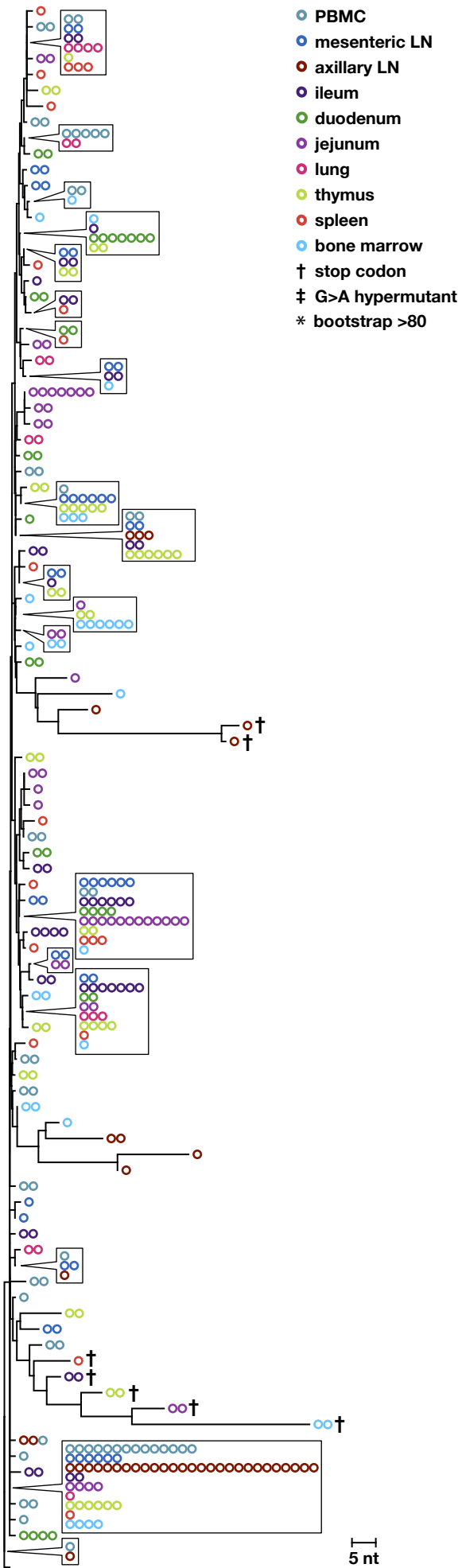

Supplement: Supplementary file 2 — 10.1186/s12977-015-0212-2 Phylogenetic relationships between single-genome proviral env sequences obtained from various anatomical compartments 30 weeks post-infection from the untreated animal 6760. The env sequence populations in the tissues (open colored circles) were not significantly different from each other indicating that the virus is well circulated across compartments, consistent with the results from the pol analyses. Highly divergent sequences are G to A hypermutants. [file 12977_2015_212_MOESM2_ESM.pdf]
